# Choosy cannibals: Targeted consumption of conspecific hatchlings by larval cane toads is triggered by species‐specific defensive toxins

**DOI:** 10.1002/ece3.8655

**Published:** 2022-03-01

**Authors:** Michael R. Crossland, Richard Shine, Jayna L. DeVore

**Affiliations:** ^1^ 4334 School of Life and Environmental Sciences A08 The University of Sydney Sydney New South Wales Australia; ^2^ 7788 Department of Biological Sciences Macquarie University Sydney New South Wales Australia

**Keywords:** Anura, behavior, *Bufo marinus*, chemical communication, embryonic development, intraspecific predation, ovivory, pheromone

## Abstract

In many species, cannibalism is uncommon and involves nonselective consumption of conspecifics as well as heterospecifics. However, within their invasive Australian range, cane toad larvae (*Rhinella marina*) specifically target and voraciously consume the eggs and hatchlings of conspecifics, often extirpating entire clutches. In contrast, toad larvae rarely consume the eggs and hatchlings of native frogs. Here, we use laboratory studies to demonstrate that this selective consumption is triggered by species‐specific chemical cues: maternally‐invested bufadienolide toxins that otherwise defend cane toad eggs and hatchlings against predators. We find that these cues stimulate feeding behaviors in toad tadpoles, such that the addition of bufadienolide toxins to the water column increases predation on eggs, not only of conspecifics, but also of native anuran species that are otherwise usually ignored. In contrast, we find that cannibalism rates on conspecific hatchlings are high and unaffected by the addition of bufadienolide cues. The maternally‐invested toxins present in conspecific eggs may therefore be more easily detected post‐hatching, at which point tadpole feeding behaviors are induced whether or not additional toxin cues are present. As bufadienolide cues have previously been found to attract toad tadpoles to vulnerable hatchlings, our present findings demonstrate that the same toxin cues that attract cannibalistic tadpoles also induce them to feed, thereby facilitating cannibalism through multiple behavioral effects. Because native fauna do not produce bufadienolide toxins, the species specificity of these chemical cues in the Australian landscape may have facilitated the evolution of targeted (species‐specific) cannibalism in invasive cane toad populations. Thus, these bufadienolide toxins confer cost (increased vulnerability to cannibalism in early life‐stages) as well as benefit (reduced vulnerability to predation by other taxa).

## Graphical abstract

Bufadienolide toxins both attract cannibalistic cane toad tadpoles to vulnerable younger conspecifics and induce feeding behavior.

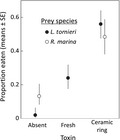

## 1 INTRODUCTION

Intuition suggests that cannibalism should be rare in nature, because an individual that consumes one of its relatives may thereby reduce its inclusive fitness and/or become infected with species‐specific pathogens (Dawkins, 1976; Pfennig et al., 1998; Pizzatto & Shine, 2011). Nonetheless, cannibalism is widespread, although typically only a minor contributor to overall rates of mortality within a population (Elgar & Crespi, 1992; Pereira et al., 2017; Richardson et al., 2010). The victims of cannibalism are usually smaller, weaker individuals and (in at least some cases) are likely to be only distantly related to the cannibal (Pfennig, 1999). In most examples that have been described, cannibalism occurs in carnivorous species (that hence have trophic structures adapted to killing and consuming prey) and tends to be facultative. That is, predators that consume a wide variety of prey types do not refrain from including conspecifics within the diet (Caldwell & de Araujo, 1998; Mettouris & Giokas, 2017; Polis & Myers, 1985). Such cases of incidental cannibalism may reflect errors in prey identification, or benefits related to energy acquisition, reduction in population density, and removal of infected individuals (Babbitt & Meshaka, 2000; Elgar & Crespi, 1992). More interesting, however, are cases where cannibalism is common in species that do not usually consume animal prey. In these cases, cannibalism may be favored because it reduces intraspecific competition for limited resources (Vijendravarma et al., 2013). For example, the juveniles of many herbivorous insects benefit from consuming smaller conspecifics (Richardson et al., 2010).

Cannibalism is widespread among the tadpoles of anuran amphibians, even in species where larvae generally graze on vegetative matter rather than consuming animal prey (Crump, 1983, 1992; Hawley, 2009; Kuzmin, 1991). Many cases involve facultative consumption of smaller conspecific tadpoles in ephemeral waterbodies, where a shift from herbivory to cannibalism can accelerate development (Babbitt & Meshaka, 2000; Heinen & Abdella, 2005; Pfennig, 1990, 1992) and, thus, allow metamorphosis prior to pond‐drying (Crump, 1983, 1992) or enable continued development despite severe food limitation (Kuzmin, 1991; McCallum & Trauth, 2001). Larvae of many amphibian species also consume conspecific eggs, thereby obtaining nutrition and reducing intraspecific competition (Crossland, Hearnden, et al., 2011; Crump, 1992).

One system in which cannibalism is common is within invasive populations of the cane toad (*Rhinella marina*) in tropical Australia. Tadpoles consume conspecific eggs and hatchlings (Alford et al., 1995; Crossland, Hearnden, et al., 2011; DeVore, Crossland, & Shine, 2021; DeVore, Crossland, Shine, & Ducatez, 2021), and larger terrestrial‐stage metamorphs consume smaller ones (Pizzatto & Shine, 2008). At these life‐history stages, cannibalism can be remarkably common: some metamorphs feed almost entirely on conspecifics (Pizzatto & Shine, 2008), and predation by tadpoles can eliminate >99% of eggs laid in natural waterbodies before they reach the tadpole stage (Alford et al., 1995; DeVore, Crossland, & Shine, 2021). Remarkably, targeted cannibalism of developing eggs and hatchlings by cane toad larvae has evolved in invasive populations subsequent to the species’ translocation to Australia; this behavior is rare within the cane toad's native range in South America (DeVore, Crossland, Shine, & Ducatez, 2021). As an adaptation that facilitates the elimination of conspecific clutches laid in their breeding pond (i.e., potential future competitors), toad tadpoles from invasive Australian populations have evolved the ability to detect these clutches via chemical cues that exude from egg clutches post‐hatching; larvae vigorously seek out and consume clutches at this time (Crossland & Shine, 2011; DeVore, Crossland, & Shine, 2021; DeVore, Crossland, Shine, & Ducatez, 2021). The chemical cues that elicit attraction are toxins (bufadienolides) that are allocated to the egg during vitellogenesis (Crossland et al., 2012, 2021; Hayes et al., 2009). Cannibalistic behavior by cane toad tadpoles is apparently widespread and consistent across invasive populations within Australia. For example, using the offspring of adult toads collected across Australia, DeVore, Crossland, & Shine (2021) found little variability in (a) the attraction response to conspecific hatchlings (24 tadpole clutches: 11 QLD, 1 NT, 11 WA, 1 NSW; 10 hatchling clutches: 2 QLD, 4 NT, 4 WA; in 56 combinations) or (b) the consumption of conspecific hatchlings (41 tadpole clutches: 22 QLD, 3 NT, 13 WA, 3 NSW; 18 hatchling clutches: 7 QLD, 4 NT, 7 WA; in 91 combinations). The strong attraction to vulnerable conspecifics can facilitate targeted cannibalism in breeding ponds by bringing cannibalistic tadpoles in close proximity to their prey. However, tadpoles from invasive Australian populations also cannibalize hatchlings more quickly than do native‐range tadpoles when the tadpoles and hatchlings are held in close proximity (DeVore, Crossland, Shine, & Ducatez, 2021). This disparity implies that this attraction is not the only behavioral difference between native and invasive cane toad populations; invasive range tadpoles are also more likely to consume vulnerable conspecifics once they reach them. However, it is unclear whether chemical cues could also play a role in stimulating this cannibalistic feeding response.

Cane toads in Australia therefore provide a unique study system in which cannibalism is newly‐evolved but very frequent (see above) and also is highly targeted. In contrast, the eggs and hatchlings of native frogs, although similar to toad eggs and hatchlings in size and structure, are apparently rarely consumed by cane toad larvae (Crossland, 1998). How is this species specificity achieved? One possibility is that the same bufadienolide toxins that attract Australian cane toad tadpoles to conspecific hatchlings also elicit feeding behavior. These cues are lacking in eggs and hatchlings of native Australian frog species, none of which belong to the family Bufonidae. Alternatively, the preference for feeding on conspecific eggs and hatchlings might be mediated by other chemical cues, or by physical characteristics of the eggs and hatchlings themselves. To establish whether toad tadpoles preferentially consume conspecifics, we first compared rates of predation by toad tadpoles on the eggs and hatchlings of native frogs to those on conspecifics. We then tested whether the presence of toxin cues intensifies cannibalism by exposing cane toad eggs and hatchlings to cannibalistic cane toad tadpoles in the absence versus presence of additional bufadienolide cues. To test whether the absence of these cues in native frog species explains the lack of predation by cane toad tadpoles on native frog eggs, we also exposed the eggs of a native frog species (black‐shinned rocket frog, *Litoria tornieri*) to cane toad tadpoles in the absence versus presence of bufadienolide cues. If the bufadienolide cues that are known to stimulate attraction also stimulate feeding, we expect the eggs of cane toads and the native frog to be consumed at higher rates when bufadienolide cues are added to the water column. In contrast, we do not expect consumption of cane toad hatchlings to be affected by the addition of bufadienolide cues, because these hatchlings already release toxin cues into the surrounding water (to which cannibal tadpoles vigorously respond; DeVore, Crossland, & Shine, 2021; DeVore, Crossland, Shine, & Ducatez, 2021).

## 2 MATERIALS AND METHODS

The cane toad (*Rhinella marina*; *Bufo marinus* in earlier literature) is a large (to >1 kg) bufonid anuran native to South America that was introduced to Australia in 1935 as a biocontrol agent and has since spread across much of the Australian continent (Shine, 2010, 2018). Female toads produce large clutches (>10,000 eggs: DeVore, Crossland, & Shine, 2021) in lentic waterbodies, in long strings embedded within gelatinous material and typically wound around aquatic vegetation (Lever, 2001). The eggs hatch within a few days (depending on water temperature: Lever, 2001). Toad tadpoles consume such eggs, but the highest rate of cannibalism is thought to fall on hatchlings that have completed embryogenesis but are not yet able to swim (DeVore, Crossland, & Shine, 2021). Toad tadpoles do not consume conspecifics after they have reached the mobile free‐swimming tadpole stage (Gosner (1960) stage 25: DeVore, Crossland, & Shine, 2021).

### 2.1 Husbandry and experimental procedures

Adult cane toads were collected from the Adelaide River floodplain and housed at the nearby Tropical Ecology Research Facility, Northern Territory (12°34′43.54″S, 131°18′51.55″E) in outdoor bins (1 m × 1 m × 0.8 m) with refugia, water, and constant food supply. We induced toads to spawn by subcutaneous injection of synthetic gonadotrophin leuprorelin acetate (Lucrin, Abbot Australasia; 0.25 mg ml^−1^). Male toads were injected with 0.25 ml and female toads with 0.75 ml as per previous studies (Crossland et al., 2021; DeVore, Crossland, & Shine, 2021; DeVore, Crossland, Shine, & Ducatez, 2021). Pairs of toads were placed in 80 L plastic tubs with a small amount of water and allowed to spawn overnight. The resultant eggs were transferred to 18 L plastic tubs filled with 9 L water, constantly aerated. Once embryos developed into free‐swimming tadpoles (Gosner stage 25), they were transferred to outdoor 750 L bins and fed algae wafers (Hikari, Kyorin, Japan) with weekly water changes. Cane toad eggs, hatchlings, and tadpoles were haphazardly selected for experiments, as required. In total, we used four toad tadpole clutches and three toad egg clutches in our experiments. The highly consistent cannibalistic behavior of cane toad tadpoles to conspecific eggs/hatchlings (DeVore, Crossland, & Shine, 2021) allowed us to minimize the number of toad clutches used, as required by University ethics guidelines. For the experiment testing predation on native anuran embryos, a single naturally‐laid clutch of *L*. *tornieri* was collected from a pond on the Adelaide River floodplain. At the completion of experiments, cane toad tadpoles were euthanized (Tricaine Methanesulfonate, MS222) according to ethics guidelines. Native frog tadpoles were released at the site of collection.

### 2.2 Experiment 1: Toad tadpoles as predators of frog embryos versus toad embryos

Previous studies investigating toad tadpoles as predators of either conspecific (Alford et al., 1995; Crossland, Hearnden, et al., 2011; DeVore, Crossland, & Shine, 2021; DeVore, Crossland, Shine, & Ducatez, 2021) or native frog (Crossland, 1998) embryos have used different experimental methodologies and statistical analyses, making formal comparison difficult. To address this issue, we extracted data for predation by cane toad tadpoles on embryos of five native frog species (*Cyclorana* [formerly *Litoria*] *alboguttata*, *Cyclorana brevipes*, *Litoria gracilenta*, *Litoria rubella*, *Platyplectrum ornatum* [formerly *Limnodynastes ornatus*]) from experiments detailed in Crossland (1998). Briefly, predation was assessed in laboratory experiments (air temperature 23–26°C) using 440 ml containers filled with 350 ml water. For each native anuran species, 10 eggs (stage 10–11) from a single clutch were added to each of 20 containers. Ten containers were randomly chosen as the predation treatment and had one toad tadpole (stage 28–40) added. The remaining 10 containers served as controls. The number of embryos surviving at the free‐swimming tadpole stage (stage 25; ~72**–**96 h later, depending on species) and the number consumed (as opposed to mortality by other causes such as developmental failure) were recorded for each container; predation data in these experiments are therefore the number of eggs and hatchlings consumed, combined. We replicated this methodology in the present study to quantify predation by cane toad tadpoles on conspecific embryos under the same experimental conditions (i.e., 1 toad tadpole clutch, 1 toad egg clutch, toad eggs stage 10–11 at the start of the experiment, 440 ml containers filled with 350 ml water, *N* = 10 replicates per treatment, air temperature 25°C; predation assessed ~72 h later). All experiments stopped when embryos developed into free‐swimming stage 25 tadpoles because cane toad tadpoles do not attack mobile prey (Crossland, 1998; DeVore, Crossland, & Shine, 2021).

#### 2.2.1 Experiment 1: Statistical analyses

We first combined the data for the five native frog species to address the question: overall, does the presence of a toad tadpole significantly affect the survival of native frog eggs? The analysis used fixed effects of treatment (toad tadpole absent vs. present), frog species, and treatment x frog species, with a temporal block (i.e., date; *C*. *brevipes*, *C*. *alboguttata*: January 1994; *L*. *gracilenta*, *L. rubella*, *P*. *ornatum*: March 1994; *R. marina*: March 2016) as a random effect. The treatment x frog species interaction was not significant (χ^2^ = 2.69, *df* = 4, *p* = .61) and so was removed from the final model. We retained the main effect of native frog species in the final model to estimate treatment effects. To examine the response for each frog species individually, we first assessed the survival of embryos to stage 25 as a response to the fixed effect of predation treatment (toad tadpole absent vs. present). These analyses addressed the question: does the presence of a toad tadpole significantly reduce the survival of the focal prey species? To specifically compare predation rates on native frog embryos versus toad embryos, we then analyzed predation (number of eggs/hatchlings consumed) by toad tadpoles within the predator‐exposed treatment as a response to the fixed effect of prey species (native frogs vs. toad), while including temporal block (i.e., date, as described above) in the model as a random effect and conspecific prey as the reference group. For these and all subsequent analyses, we analyzed data in R (R Core Team, 2021) as a binomial response to treatment (control vs. exposed treatments: alive, dead; predator‐exposed treatment: not eaten, eaten) using logistic regression (Warton & Hui, 2011) and quasi‐binomial models to account for data under‐dispersion or over‐dispersion (fixed effects only models: glm; mixed‐effects models: pooled native frog data package MASS:glmmPQL (Venables & Ripley, 2002) followed by package car:Anova (Fox et al., 2020), all other mixed‐effects models package MASS:glmmPQL (Venables & Ripley, 2002)). Data for *L*. *gracilenta*, *L. rubella*, *P*. *ornatum*, and *C*. *brevipes* were under‐dispersed; all other data in this and subsequent experiments were over‐dispersed. The models for predation treatment effects for *C*. *alboguttata*, *P*. *ornatum*, and *R. marina* could not reach convergence because survival in all control treatment containers was 100%. In these instances, we assigned one individual in one control container to have died, to obtain a conservative estimate of treatment effects (Warton & Hui, 2011).

For these and all subsequent analyses, we calculated the positive and negative standard error values of mean estimates by both adding and subtracting the model output SE value from the model output effect size estimate (all on the logit scale), then exponentiating these values to transform them into odds ratios. As a result, the SE values are unevenly distributed around the mean, and are represented by a SE interval. For example, if the model output gave results on the logit scale of Estimate = 2.7515 and SE = 0.5208, then the mean odds ratio was calculated by exponentiating 2.7515, the lower SE estimate was calculated by exponentiating (2.7515 − 0.5208), and the upper SE estimate was calculated by exponentiating (2.7515 + 0.5208). In this example, conversion from the logit scale gives an odds ratio (SE interval) of 15.7 (9.3, 26.4). Where appropriate, these odds ratio values were converted to proportions by using the formula (value / 1 + value). That is, proportion mean = 15.7 / (1 + 15.7) = 0.94, proportion lower SE = 9.3 / (1 + 9.3) = 0.90, proportion upper SE = 26.4 / (1 + 26.4) = 0.96.

### 2.3 Experiment 2: Effect of bufadienolide cues on cannibalism of toad eggs and hatchlings

To assess the effect of additional bufadienolide cues on cannibalistic behavior of cane toad tadpoles, we measured cannibalism rates in the absence versus presence of these cues. Bufadienolides were obtained from the frozen parotoid glands of adult toads (Chen et al., 2017); this parotoid toxin, like the toxin present in toad eggs and hatchlings, contains a diverse mixture of bufadienolide chemicals (Crossland et al., 2021; Hayes et al., 2009). Some of the chemicals present in frozen parotoid glands do not occur in eggs (bufalin, resibufagin), whereas other chemicals present in eggs do not occur in frozen parotoid glands (bufolipin A, unspecified bufolipins and bufagenins: Crossland et al., 2021). However, several bufadienolides occur in frozen parotoid glands, eggs, and hatchlings (marinobufagin, telocinobufagin, hellebrigenin), with marinobufagin being the major component of the toxin profile of both frozen parotoid glands and eggs (Crossland et al., 2021), as well as a significant component of the toxin profile of hatchlings (Hayes et al., 2009). Both marinobufagin and adult toad parotoid gland toxin secretion strongly attract cane toad tadpoles (Crossland et al., 2012, 2021). Furthermore, attraction responses to bufadienolides extracted from frozen parotoid glands are equivalent to those elicited by bufadienolides extracted from eggs (Crossland et al., 2021). For these reasons, we considered bufadienolides extracted from frozen parotoid glands to be a realistic proxy for cane toad egg/hatchling toxin cues for the purpose of examining cannibalistic responses of toad tadpoles.

For our trials, 2 mg of bufadienolides was extracted from parotoid secretions as described by Crossland et al. (2021). Briefly, frozen toads (stored at −20°C) were thawed, and parotoid glands (54 g) were macerated in 250 ml water with a commercial blender, then filtered through a bed of Celite 545. The filtrate was concentrated in vacuo at 40°C and partitioned into ethyl acetate and water solubles. The ethyl acetate extract containing mostly bufagenins was used without further purification. A stock solution was prepared in MeOH (10 mg/ml) with a fixed volume (0.2 ml) loaded onto porous ceramic rings (Majestic Aquariums, Sydney, NSW) to give a loading of 2.0 mg extracted bufadienolides per ceramic ring. Control ceramic rings were loaded with 0.2 ml MeOH. All rings were left in a fume hood overnight at room temperature to allow the MeOH to evaporate. Approximately 70% of the extract in toxin rings was marinobufagin (unpubl. data). Control rings were left toxin‐free. Alone, these ceramic rings are biologically inert and do not initiate cannibalistic attraction or feeding behavior in toad tadpoles (Crossland et al., 2021). Bufadienolides leech from toxin‐embedded ceramic rings to cause significant attraction behavior by cane toad tadpoles within 1–2 h (Crossland et al., 2021).

We added 10 *R. marina* eggs (stage 5–6) from a single clutch to each of 21 × 1 L containers filled with 750 ml water (temperature 26°C). These treatment bins were arranged in seven spatial blocks. Within each block, containers were randomly allocated to one of three treatments: (1) control, (2) one toad tadpole plus control ceramic ring, or (3) one toad tadpole plus toxin ceramic ring (*N* = 7 replicates). The toad eggs and tadpoles used in this experiment were each sourced from separate clutches, and different from the clutches used in Experiment 1. We recorded survival and number of individuals consumed at intervals throughout egg and hatchling development. We stopped the experiment once the embryos had developed into free‐swimming tadpoles (stage 25).

#### 2.3.1 Experiment 2: Statistical analyses

The purpose of treatment 1 (control) was to identify the level of background mortality not related to cannibalism. Because survival in this treatment was 100%, we focused our analyses on toxin treatment effects when *R. marina* eggs and hatchlings were exposed to a toad tadpole (i.e., treatment 2 vs. treatment 3). In these treatments, all mortality was due to cannibalism. To assess the effects of toxin cues on cannibalism, we first examined toxin treatment effects (bufadienolide cues absent vs. present) using the entire data set to stage 25 (i.e., cannibalism on egg and hatchling stages, combined). Because the effects of adding toxin may differ depending on whether or not the eggs had hatched (for example, if the maternally‐invested toxins present in the eggs are only detectable post‐hatching), we then assessed the effects of adding toxin cues on cannibalism on eggs (≤stage 17) and hatchlings (≥stage 18) separately. For analyses of egg cannibalism, we included all individuals added to containers as eggs at the start of the experiment. For analyses of hatchling cannibalism, we only considered individuals that were still alive at the end of the egg stage (~36 h later; i.e., all individuals consumed as eggs before this time were excluded from the hatchling analysis).

Analyses were conducted using fixed effects of treatment, time, and treatment x time, using a random slope model in which tub and spatial block were included as nested random effects. Time (measured in hours) was included as a numeric predictor in the model. For example, in the egg/hatchling cannibalism trial, the first survival check was conducted 1 h after the cannibalistic tadpole was introduced (Time = 1). Subsequent survival checks were done hourly during daylight hours until hatchlings reached the tadpole stage (stage 25, 51 h later, Time = 51). The inclusion of tub as a random effect allowed us to account for these repeated measures, and the random slope model allowed us to account for variation between individual tadpoles in the rate at which they cannibalized conspecifics. The proportion of survivors in each tub at each monitoring period was taken as the response variable.

When the treatment x time interaction term was nonsignificant, it was removed and we re‐ran the model. This meant that the interaction term was removed for the overall cannibalism data model (*t* = 0.66, *df* = 152, *p* = .51) and the hatchling cannibalism model (*t* = 0.80, *df* = 54, *p* = .43), but retained for the egg cannibalism model (*t* = 2.40, *df* = 82, *p* = .019; see Results for further details). In instances where the treatment × time interaction was removed, we retained both treatment and time as fixed effects in the final model. For estimation of toxin treatment effects at egg stage 14 (~22 h), the model could not reach convergence because no eggs had yet been cannibalized in any of the control treatment containers. In this instance, we assigned one individual in one control container to have been eaten, to obtain a conservative estimate of treatment effects on cannibalism (Warton & Hui, 2011). Adjustments were not required for any other models.

The addition of toxin cues caused increased cannibalism of eggs but not hatchlings (see Results below). This lack of treatment effect on hatchling cannibalism could be due to hatchlings releasing their own toxin into the surrounding water after emerging from egg strings (DeVore, Crossland, & Shine, 2021; DeVore, Crossland, Shine, & Ducatez, 2021), thus making additional toxin cue ineffective in terms of changing behavior of cannibal tadpoles. Alternately, hatchling consumption in the toxin‐addition treatment may have been reduced simply because the toad tadpoles were satiated after consuming eggs. To address this issue, we re‐ran the protocol above using different clutches of both toad eggs and toad tadpoles (i.e., 1 new egg clutch, 1 new tadpole clutch), this time starting the experiment with 10 stage 18 hatchlings in the place of 10 unhatched eggs. Seven replicate bins were used per treatment, and hatchling cannibalism was checked hourly throughout the day (with 10–12 h breaks per night). Survival in the control treatment was 100%. Thus, we removed this treatment from analysis to focus on the effect of toxin treatment on hatchling cannibalism. As per the previous experiment, analyses were conducted using fixed effects of treatment, time, and treatment x time, using a random slope model in which tub and spatial block were included as nested random effects (see above for further details). The nonsignificant treatment x time interaction term (*t* = 1.15, *df* = 418, *p* = .25) was removed from the final model, while both treatment and time were retained as fixed effects.

### 2.4 Experiment 3: Effect of bufadienolide cues on predation of frog eggs

To assess whether bufadienolide cues affect the predatory response of toad tadpoles to native frog eggs, we measured predation rates on native frog eggs in the absence versus presence of these cues. In these trials, we tested toad tadpole responses to two bufadienolide treatments. For one treatment, 100 mg toxin was gently squeezed from the parotoid glands of live toads onto a glass slide and then placed in experimental containers. Based on the composition of cane toad parotoid gland exudates, this secretion would have contained a mixture of ~1 mg bufadienolide chemicals. For the second treatment, we used a 2 mg mixture of bufadienolide chemicals loaded onto ceramic rings (as described in Experiment 2). Both treatments would have had the same toxin composition, with ~70% of the bufadienolide mixture being marinobufagin (i.e., ~0.7 mg marinobufagin in the fresh toxin treatment and ~1.4 mg marinobufagin in the ceramic ring treatment; unpubl. data).

We added 10 *L*. *tornieri* eggs (stage 10–11) to each of 25 × 1 L containers filled with 750 ml water. Containers were randomly allocated to one of five treatments: (1) control, (2) 1 toad tadpole, (3) 1 toad tadpole + control ceramic ring, (4) 1 toad tadpole + ~1 mg fresh bufadienolide mixture, or (5) 1 toad tadpole + 2 mg bufadienolide ceramic ring (*N* = 5 replicates); these treatments were placed in five spatial blocks, within which their position was randomized. Toad tadpoles were sourced from a single clutch, different from the clutches used in Experiments 1 and 2. We recorded embryo survival and number of eggs consumed until the eggs reached stage 23, which for *L*. *tornieri* is the stage immediately prior to hatchlings emerging from egg capsules. We stopped the experiment at this stage because free‐swimming native anuran tadpoles are sensitive to toad toxin in solution (Crossland, Brown, & Shine, 2011), although the stage 23 *L*. *tornieri* embryos within their egg capsules were apparently unaffected and developed normally.

#### 2.4.1 Experiment 3: Statistical analyses

Mean survival in the control treatment (treatment 1: eggs only) was 99.3% (SE interval: 97.9%, 99.8%). Therefore, we focused our analyses on treatment effects where *L*. *tornieri* eggs were exposed to a toad tadpole. The purpose of treatment 2 (1 toad tadpole) versus treatment 3 (1 toad tadpole + control ceramic ring) was to determine whether the presence of an inert ceramic ring alone affected predation on *L*. *tornieri* eggs; it did not (mean proportion of eggs eaten in both treatments 2 and 3 = 0.02, (SE interval: 0.01, 0.05); *t* = 0.00, *df* = 9, *p* = 1.00). Therefore, we also excluded treatment 2 from analyses. To assess the effect of toxin cues on predation of frog eggs by cane toad tadpoles (treatments 3, 4, 5), we included the fixed effect of predator tadpole treatment (ceramic control ring, fresh toxin, or ceramic toxin ring) with spatial block as a random effect. To determine whether differences between toxin treatments could be attributable to the greater toxin concentrations in the toxin ring treatment, we then re‐ran this analysis with bufadienolide concentration (0, 1 or 2 mg) included as a continuous predictor of frog egg predation (in place of the categorical treatment predictor).

### 2.5 Experimental comparisons

In Experiment 1, we found that toad tadpoles were far more likely to consume eggs/hatchlings of conspecifics (combined data) than those of native frogs (see Results). However, the data collected in Experiment 1 did not allow us to assess predation on egg and hatchling stages separately. Thus, it was unclear whether predation rates also differ between conspecifics and heterospecifics within the egg stage (prior to hatching). To determine whether the rate of consumption of unhatched eggs of the native frog *L*. *tornieri* differs from consumption of conspecific eggs, or if the addition of toxin influences these effects, we therefore compared predation on unhatched embryos immediately prior to hatching for each species (i.e., at Gosner stage 17 for *R. marina* and stage 23 for *L*. *tornieri*). To make this comparison, we used the *L*. *tornieri* data described above and the stage 17 (36 h exposure) *R. marina* data from the cannibalism experiment described in Experiment 2. Therefore, exposure to a predatory tadpole lasted for 11–12 Gosner stages for *R. marina* (Gosner stage 5–6 to 17; 36 h) and 13 Gosner stages for *L*. *tornieri* (Gosner stage 10–23; 63 h). The effect of prey species on egg predation was then compared both in control conditions (i.e., in the presence of an inert ceramic ring) and in the presence of added toxins (i.e., in the presence of a ceramic ring containing 2 mg of bufadienolides) using separate binomial models in which prey species was included as a fixed effect.

## 3 RESULTS

### 3.1 Experiment 1: Toad tadpoles as predators of frog embryos versus toad embryos

Overall, the presence of a toad tadpole significantly reduced the survival of frog eggs (combined native frog data, control vs. predator‐exposed treatment: χ^2^ = 6.79, *df* = 1, *p* = .009). However, this treatment effect was biologically small (mean proportion survival (SE interval): control = 0.992 (0.984, 0.996) vs. predator‐exposed = 0.949 (0.929, 0.963)). The main effect of native frog species was not significant (χ^2^ = 3.98, *df* = 4, *p* = .41).

When analyzed individually, the presence of a toad tadpole did not significantly reduce survival of any of the native species (control vs. predator‐exposed treatment; mean proportion survival (SE interval): *C*. *alboguttata t* = −1.23, *df* = 19, *p* = .24, control = 0.989 (0.939, 0.998) vs. exposed = 0.899 (0.827, 0.943); *C*. *brevipes t* = −2.01, *df* = 19, *p* = .06, control = 0.989 (0.975, 0.996) vs. exposed = 0.930 (0.902, 0.950); *L*. *gracilenta t* = −0.01, *df* = 19, *p* = .99, control = 0.989 (0.973, 0.996) vs. exposed = 0.989 (0.973, 0.996); *L. rubella t* = 0.58, *df* = 19, *p* = .57, control = 0.979 (0.961, 0.989) vs. exposed = 0.989 (0.974, 0.996); *P*. *ornatum t* = −1.75, *df* = 19, *p* = .10, control = 0.989 (0.974, 0.996) vs. exposed = 0.940 (0.912, 0.959)). However, the presence of a toad tadpole did significantly reduce the survival of conspecifics (*t* = −5.45, *df* = 19, *p* < .0001, control = 0.989 (0.966, 0.997) vs. exposed = 0.060 (0.037, 0.097)).

**FIGURE 1:**
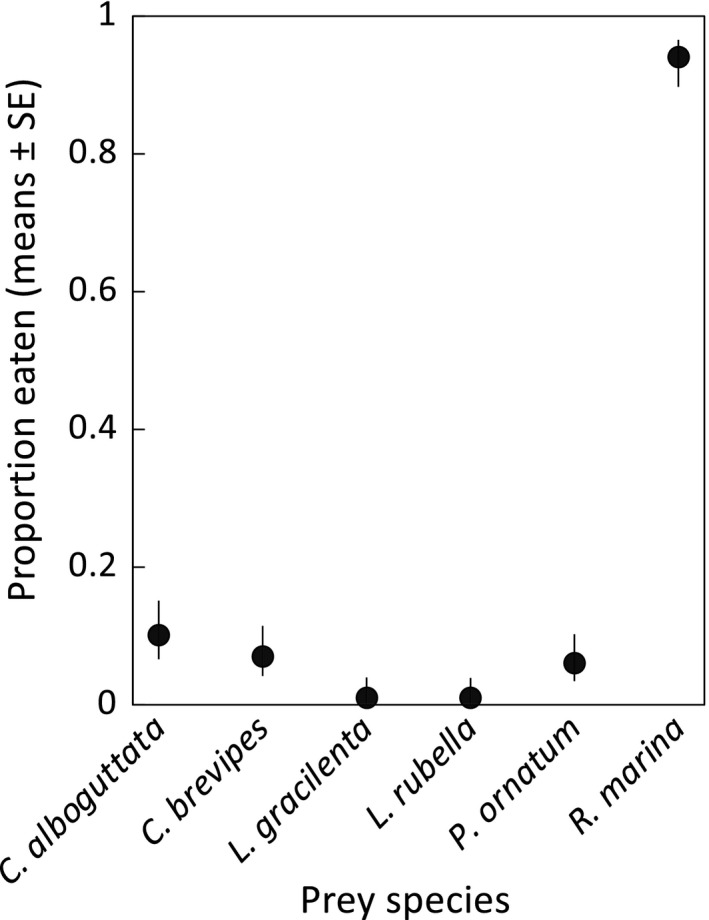
Comparison of cane toad tadpoles as predators of native frog eggs/hatchlings versus conspecific eggs/hatchlings under standardized experimental conditions. Ten individuals were exposed to predation by a single toad tadpole for each trial (for 10 replicate trials per species); data depict the proportion of individuals eaten before they could develop into free‐swimming tadpoles (Gosner (1960) stage 25), at which point they are no longer vulnerable to predation by cane toad tadpoles. Data on native frogs were extracted from Crossland (1998)

Comparison among the exposed treatments showed that cane toad tadpoles consumed fewer eggs/hatchlings of each native frog species compared to those of conspecifics (*C*. *alboguttata t* = −6.65, *df* = 52, *p* < .0001; *C*. *brevipes t* = −6.71, *df* = 52, *p* < .0001; *L*. *gracilenta t* = −4.86, *df* = 52, *p* < .0001; *L. rubella t* = −4.88, *df* = 52, *p* < .0001; *P*. *ornatum t* = −6.68, *df* = 52, *p* < .0001; Figure 1).

### 3.2 Experiment 2: Effect of bufadienolide cues on cannibalism of toad eggs and hatchlings

**FIGURE 2:**
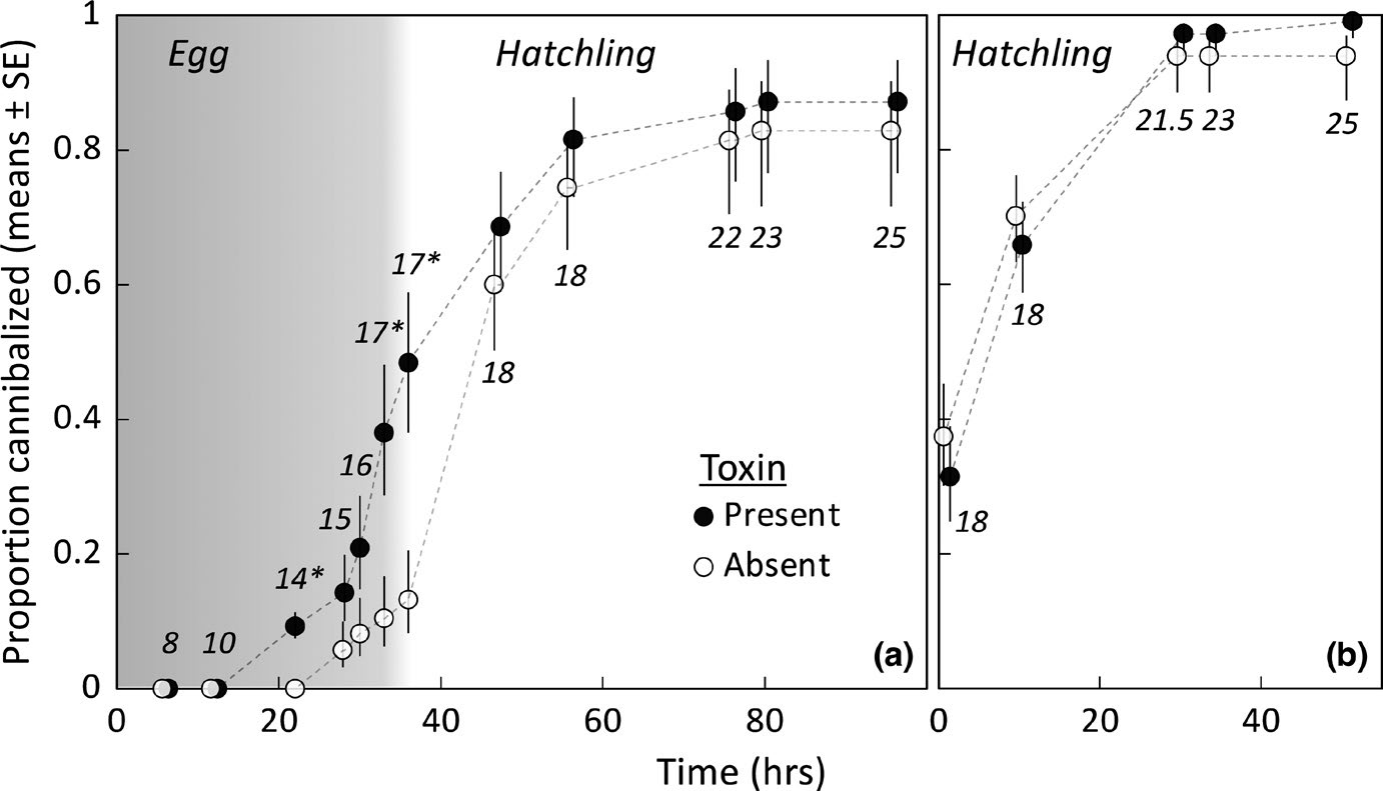
The proportion of 10 cane toad embryos or hatchlings exposed to a single cane toad tadpole that had been cannibalized at each monitoring period. Cannibalism rates were measured in the presence of a ceramic ring that either contained 2 mg of bufadienolide toxins (treatment: toxin present, *N* = 7) or did not contain toxins (treatment: toxin absent, *N* = 7). The numbers associated with each period indicate the mean developmental stage (Gosner, 1960) of the monitored individuals. In panel a experimental exposure to the cannibal tadpole began at Gosner stage 5–6 and hatching occurred late in stage 17; egg stages are shown in the gray section and hatchling stages in the white section. In panel b exposure to a cannibal tadpole began at hatchling stage 18. Monitoring in both experiments ended at stage 25; at this point, hatchlings have become free‐swimming tadpoles and can no longer be cannibalized. Asterisks indicate stages at which significantly more individuals had been cannibalized in the toxin treatment (*p* < .05). In the absence of a cannibalistic cane toad tadpole, survival to Gosner stage 25 was 100% (data not shown)

There was a significant effect of toxin treatment on the proportion of toad eggs and hatchlings (combined data) that were cannibalized prior to reaching the free‐swimming tadpole stage (*t* = 2.82, *df* = 6, *p* = .03). Overall, the odds that eggs/hatchlings would be consumed in the presence of toxin bait cues were 3.31 times (SE interval: 2.17, 5.07) those in the absence of these cues (Figure 2a). The proportion of eggs/hatchlings consumed by conspecific tadpoles also increased through time (*t* = 10.00, *df* = 153, *p* < .0001; Figure 2a).

Separate analyses of the egg and hatchling stages revealed differences in the effects of the toxin treatment on cannibalism. Within the egg stage (time 6–36 h post‐exposure, Gosner stages 8–17), there was a significant treatment x time interaction (*t* = 2.40, *df* = 82, *p* = .019). Therefore, for egg cannibalism, we analyzed treatment effects at each time interval separately. There was an initial period before egg cannibalism commenced in either treatment (time 6–12 h, stages 8–10; Figure 2a); thereafter, cannibalism began earlier in the toxin treatment than the control (time 22 h, stage 14; *t* = 3.76, *df* = 6, *p* = .01; Figure 2a). At this time, the odds that an egg would be eaten in the toxin treatment were 4.75 times (SE interval: 3.14, 7.19) those in the control treatment. Beginning at time 28 h (stage 15), egg cannibalism had also occurred in the control treatment, such that egg consumption did not differ significantly between treatments at 28 or 30 h (stage 16) post‐exposure (respectively: *t* = 1.40, *df* = 6, *p* = .21; *t* = 1.75, *df* = 6, *p* = .13; Figure 2a). However, by 33 and 36 h (stage 17), significantly more eggs had been consumed in the toxin treatment (respectively: *t* = 2.98, *df* = 6, *p* = .025; *t* = 3.319, *df* = 6, *p* = .02; Figure 2a). At 33 h, the odds that eggs would be cannibalized in the presence of toxin cues were 5.26 times those in the absence of toxin cues (SE interval: 3.01, 9.17), and by 36 h this odds ratio was 6.14 (SE interval: 3.56, 10.63).

Within the hatchling stage (≥stage 18), there was an effect of time on proportion of hatchlings consumed (*t* = 5.27, *df* = 55, *p* < .0001) but no significant effect of toxin cue treatment (*t* = −1.67, *df* = 6, *p* = .15; Figure 2a). That is, among individuals that had survived to the hatchling stage, the proportion that was subsequently cannibalized did not differ significantly between toxin present versus absent treatments. The effect of toxin addition on overall cannibalism rates (combined egg and hatchling data) was thus due to tadpoles being more likely to consume conspecifics during the egg stage, but not the hatchling stage, if toxin was present.

Our follow‐up test of cannibalism on hatchlings (≥ stage 18) using toad tadpoles that had not previously consumed conspecifics confirmed the above result. Cannibalism of hatchlings increased through time (*t* = 4.22, *df* = 419, *p* < .0001; Figure 2b), regardless of toxin treatment (*t* = −0.05, *df* = 6, *p* = .96; Figure 2b). Hence, the lack of effect of additional bufadienolide cues on hatchling cannibalism in the previous experiment was not due to satiation of cannibal tadpoles that had consumed conspecific eggs.

### 3.3 Experiment 3: Effect of bufadienolide cues on predation of frog eggs

**FIGURE 3:**
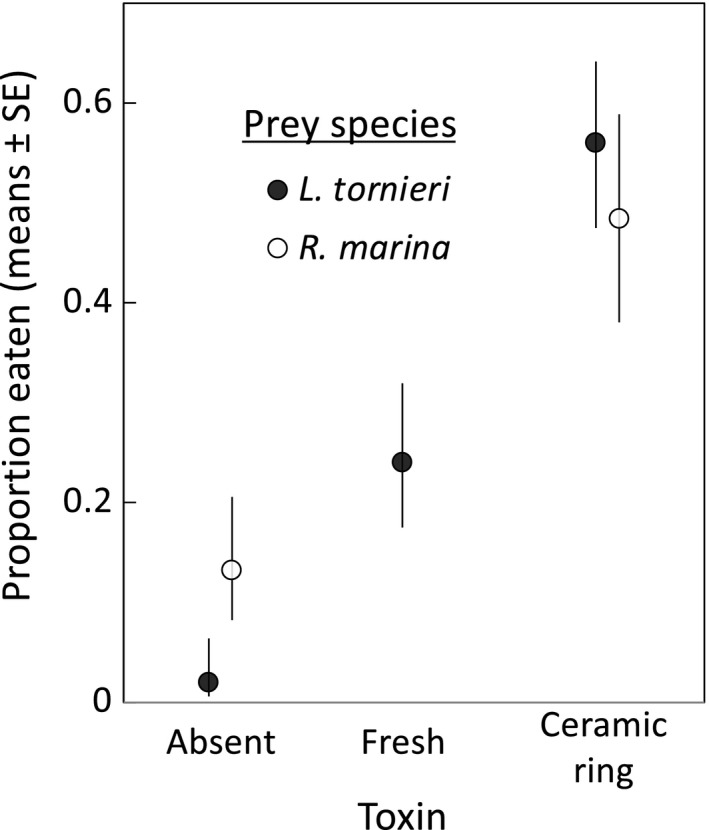
Predation by a cane toad tadpole on 10 unhatched heterospecific or conspecific embryos (*Litoria tornieri* or *Rhinella marina*) in the absence or presence of additional toxin cues (bufadienolides). Toxin treatments were either toxin freshly squeezed from adult toad parotoid glands (~1 mg bufadienolides) or toxin extracted from adult toad parotoid gland secretions and loaded onto ceramic rings (2 mg bufadienolides). Control (toxin absent) treatments contained a blank, biologically inert ceramic ring that contained no toxins. Embryo predation is depicted immediately prior to hatching (i.e., Gosner stage 23 for *L*. *tornieri* [63 h exposure] and Gosner stage 17 for *R. marina* [36 h exposure; see Figure 2]). In the absence of a predatory cane toad tadpole, mean survival for *L*. *tornieri* was 98% (range: 90% to 100%; data not shown)

Toad tadpoles ate more *L*. *tornieri* eggs when exposed to rings loaded with bufadienolides than control rings (*t* = 3.29, *df* = 8, *p* = .01; Figure 3). There was a similar pattern of increased predation on frog eggs for toad tadpoles exposed to fresh parotoid secretion compared to the control, but the difference was not significant (*t* = 2.15, *df* = 8, *p* = .06; Figure 3). Comparing the two toxin treatments, predation on *L*. *tornieri* eggs in the toxin ceramic ring treatment tended to be higher than in the fresh toxin treatment, although the difference was again not significant (*t* = 2.49, *df* = 4, *p* = .07; Figure 3). The stronger effects of the ring treatment on predation rates may be attributable to the greater toxin concentrations in this treatment; when bufadienolide concentration (0, 1, or 2 mg) was considered as the predictor of predation rates, higher predation rates were associated with greater toxin concentrations (*t* = 4.61, *df* = 9, *p* = .001; each 1 mg of increase in bufadienolide concentration increased the relative odds of being eaten by 5.66 times, SE interval: 3.89, 8.25).

Rates of predation by toad tadpoles on unhatched *L*. *tornieri* eggs were similar to those on unhatched conspecific eggs, both in control treatments (inert ceramic ring; *t* = 1.59, *df* = 10, *p* = .14; Figure 3) and the toxin ceramic ring treatment (*t* = −0.51, *df* = 10, *p* = .623; Figure 3). That is, predation rates on unhatched eggs were low in the absence of toxin cue and high in the presence of toxin cue, regardless of prey species.

## 4 DISCUSSION

Previous studies have shown that cane toad tadpoles utilize maternally‐invested bufonid toxins (Crossland et al., 2021; Hayes et al., 2009) to locate conspecific hatchlings (Crossland et al., 2012, 2021; Crossland & Shine, 2011; DeVore, Crossland, & Shine, 2021; DeVore, Crossland, Shine, & Ducatez, 2021). Our results demonstrate these toxin cues also trigger consumption of conspecifics. Bufadienolide toxins are present in toad eggs located within the jelly string (Crossland et al., 2021; Hayes et al., 2009), but are released into the water column only when the egg string and inner capsules are disrupted, such as by natural hatching processes (Crossland & Shine, 2011; present study). This reliance upon bufadienolides to initiate predatory behaviors means that native frog embryos (which lack bufadienolides) are rarely consumed by toad tadpoles (see below for further discussion). Our results are consistent with previous observations that exposure to cues from conspecific hatchlings causes biting/grazing behavior in cane toad tadpoles, even in the absence of food (Crossland & Shine, 2011); apparently, toad tadpoles are strongly attracted to the toxins detectable in conspecific hatchlings and, upon reaching the source of these cues, begin indiscriminately grazing. This finding clarifies a mechanistic pathway that enabled the evolution of targeted cannibalism after toads were introduced to Australia (DeVore, Crossland, Shine, & Ducatez, 2021) and helps to explain the extremely high rates of cannibalism documented in natural waterbodies within invasive populations (Alford et al., 1995; DeVore, Crossland, & Shine, 2021).

We also provide the first data to quantify differences in predation rates by cane toad tadpoles on native frogs versus toads in Australia under comparable experimental conditions and demonstrate that, across the vulnerable egg and hatchling stages, cannibalism of conspecifics far exceeds predation of heterospecific anurans. Notably, however, consumption of eggs was uncommon, both for eggs of *R. marina* and those of the native frog *L*. *tornieri*. Additional toxin cues increased rates of consumption of the eggs of both species. This finding indicates that the susceptibility of toad eggs to predation by toad tadpoles is not driven by unique features of physical structure. Instead, the egg strand masks the cues that induce predation, such that the large difference in predation rates between conspecifics and native frogs documented here (Experiment 1) is predominately attributable to the high rate at which conspecifics were cannibalized post‐hatching. Relative to these native frogs, which typically do not hatch until embryos are mobile, cane toads hatch much earlier in development. Incapable of movement until they develop into free‐swimming tadpoles, cane toad hatchlings can be readily grazed upon by conspecific tadpoles that lack adaptations for capturing mobile prey. Notably, this vulnerable hatchling stage is also when individuals exposed to cannibal cues exhibit an inducible defense: developmental acceleration that reduces the duration of the vulnerable period (DeVore, Crossland, & Shine, 2021).

Our experiments used a small number of clutches, raising the issue of the extent to which the results are likely to be broadly applicable. For our frog egg experiments, we used one egg clutch per species. It is possible that one or more of these clutches was atypical for the species in a manner that reduced predation by toad tadpoles (e.g., the egg capsules were unusually difficult for toad tadpoles to penetrate). We consider this unlikely for two reasons. Firstly, the consistent response of toad tadpoles to all six frog species tested (minimal predation in the absence of toxin cues: Figures 1 and 3) suggests it is unlikely we happened to choose an atypical egg clutch for every frog species. Secondly, the bufadienolide cues that trigger predatory responses of cane toad tadpoles (Crossland et al., 2021; Crossland & Shine, 2011; DeVore, Crossland, & Shine, 2021; DeVore, Crossland, Shine, & Ducatez, 2021; present study) are absent from the eggs and hatchlings of all native frog species, none of which belong to the family Bufonidae. Indeed, it was only when we added these toxin cues to the water column that cane toad tadpoles consumed frog eggs in significant numbers (Figure 3). Because all eggs and hatchlings of native frogs lack bufadienolides, there can be no inter‐clutch variation (i.e., clutch effects) for this critical chemical cue. For these reasons, we suspect the low predation rates on native frog eggs and hatchlings would occur for other clutches of the same species, and also for other native frog species not tested. Nonetheless, we acknowledge that further studies are required to verify this prediction.

Our experiments investigating cannibalism used a total of three egg clutches and four tadpole clutches. For cane toads, variation in cannibalism might occur via inter‐clutch variation in (a) maternal investment of toxin to eggs/hatchlings, and/or (b) the ability of tadpoles to detect and respond to bufadienolide cues. However, the available evidence suggests that even if such variation occurs, it has negligible ecological effect. Crossland and Alford (1998) and Crossland and Shine (2010) tested the toxicity of toad eggs/hatchlings to native frog tadpoles in Queensland and the Northern Territory (respectively, QLD: 4 clutches: M. Crossland unpublished data; NT: 7 clutches) and found no evidence of variation in maternal investment of toxins. Regarding response of toad tadpoles to toxin cues, Crossland et al. (2021) found minimal inter‐clutch variation in the attraction response of cane toad tadpoles (NT: 4 to 7 clutches) to baits made from conspecific eggs or bufadienolide chemicals. In an extensive series of experiments, DeVore, Crossland, and Shine (2021) and DeVore, Crossland, Shine, and Ducatez (2021) found no evidence of population effects in either cannibalistic attraction to conspecific hatchlings (24 tadpole clutches: 11 QLD, 1 NT, 11 WA, 1 NSW; 10 hatchling clutches: 2 QLD, 4 NT, 4 WA, in 56 combinations) or consumption of conspecific hatchlings (41 tadpole clutches: 22 QLD, 3 NT, 13 WA, 3 NSW; 18 hatchling clutches: 7 QLD, 4 NT, 7 WA; in 91 combinations). Given that both cannibalistic attraction and consumption by cane toad tadpoles are driven by bufadienolide toxins, the low variability in these studies suggests that any variation among clutches or populations in toxin content or the ability of toad tadpoles to respond to toxin cues has minimal effect on cannibalism responses. In addition, the fact that cannibalism rates on hatchlings in Experiment 2 did not increase when we added extra bufadienolide cues implies that cannibalistic behavior had already reached a maximum response once toad hatchlings had emerged from their egg strands, such that additional cues did not induce stronger responses. If variation in maternally‐invested toxin content between clutches strongly affects cannibalism rates, we should have seen a strong increase in cannibalism of hatchlings in Experiment 2 when we added additional bufadienolide cues to the water column (unless by chance the toad egg clutches used were exceptionally toxic, which seem unlikely as discussed above). We did not see such a response (Figure 2a,b). For these reasons, we believe our cannibalism results are likely to be broadly applicable for cane toad clutches throughout Australia.

The high rates of cannibalism in our small‐scale laboratory experiments in the absence of additional toxin cues (>80%) are consistent with rates in natural waterbodies (>99%: Alford et al., 1995; DeVore, Crossland, & Shine, 2021), and so are not an artifact of experimental design. The lack of significant predation on native frog embryos (Figure 1) was also not an artifact of container size (i.e., the use of small containers did not inhibit the predatory behavior of toad tadpoles), as evidenced by the frequent consumption of frog embryos in these containers when the appropriate cue (bufadienolide chemicals) was present (Figure 3). However, the magnitude of this predatory response may be dose‐dependent: the ceramic rings that induced the strongest predation effect (with 56% of *L*. *tornieri* embryos consumed) contained approximately twice as much bufadienolide as did the fresh toxin secretion (24% consumed; Figure 3). Hence, predation rates may vary depending on the quantity of toxin present.

It is important to note that, although we observed low rates of cannibalism on conspecific eggs in our laboratory feeding trials, cannibalism of the egg stage can be pronounced in natural ponds where toad tadpoles are abundant. In these ponds, toad tadpoles often consume entire egg clutches even before hatching occurs (DeVore, Crossland, & Shine, 2021). Because toad eggs contain bufadienolides (Crossland et al., 2021; Hayes et al., 2009), toad tadpoles encountering and grazing upon these eggs may release bufadienolides into the water, thereby attracting additional conspecific tadpoles and triggering egg cannibalism. Indeed, ceramic rings infused with toxins extracted from crushed, unhatched eggs strongly attract toad tadpoles (Crossland et al., 2021). Because our experimental design only utilized a single cannibalistic tadpole, we could not identify potential effects of bufadienolide release from damaged eggs on the formation of cannibalistic tadpole aggregations.

Species‐specific chemical cues from food items trigger feeding responses in many types of predators (e.g., Holding et al., 2016) as well as in herbivores (e.g., Sharp et al., 2015), but we are not aware of any other example where a specific cue induces consumption of conspecifics rather than heterospecific prey items. Instead, highly species‐specific predation (cannibalism without consumption of heterospecifics) often occurs in a context (such as a species‐poor community, an isolated habitat patch, or a uterus) where the potential prey items encountered by a cannibal are overwhelmingly of its own species, removing the need for discrimination between prey types (e.g., Elgar & Crespi, 1992). Nonetheless, numerous examples show that cannibalism can be directed nonrandomly toward individuals of specific phenotypes (sizes, activity levels, relatedness to the predator: see reviews in Elgar & Crespi, 1992; Fouilloux et al., 2019) within a population, and hence, it is unsurprising to discover that similar selectivity can come into play at the level of species.

The species‐specific defensive toxins that are utilized by cane toads to target conspecific hatchlings have played a key role in facilitating the success of this invasive species in Australia. Because no native Australian fauna produce bufadienolides, the predator assemblage is poorly adapted to cope with these toxins, and their ingestion is lethal to a variety of aquatic and terrestrial predators (Crossland & Alford, 1998; Crossland et al., 2008; Shine, 2010). Indeed, the efficacy of these toxins in deterring predation may have contributed to the high toad densities (Lampo & De Leo, 1998) and pronounced intraspecific competition (Crossland, Hearnden, et al., 2011) that likely favored the evolution of cannibalistic behaviors within these invasive populations (DeVore, Crossland, & Shine, 2021). Although the costs of toxic defenses are often calculated in terms of how energetically expensive they are to produce (Blennerhassett et al., 2019; Enzor et al., 2011; Harris & Jenner, 2019), here we demonstrate a strong, additional cost; the utilization of this predator defense comes at the expense of increased cannibalism risk. Whether the emergence of targeted cannibalism in the invasive range will ultimately favor reduced maternal investment in egg toxicity remains an open question for future research.

## CONFLICT OF INTEREST

There are no conflicts of interest.

## AUTHOR CONTRIBUTIONS

**Michael R. Crossland:** Conceptualization (equal); Data curation (equal); Formal analysis (equal); Investigation (equal); Methodology (equal); Writing – original draft (equal); Writing – review & editing (equal). **Richard Shine:** Conceptualization (equal); Formal analysis (equal); Funding acquisition (equal); Project administration (equal); Resources (equal); Supervision (equal); Writing – original draft (equal); Writing – review & editing (equal). **Jayna L. DeVore:** Conceptualization (equal); Formal analysis (equal); Investigation (equal); Methodology (equal); Writing – review & editing (equal).

## Data Availability

Data have been deposited in Dryad https://doi.org/10.5061/dryad.pc866t1qm.
